# CRISPR/Cas9-mediated mutation of tyrosinase (Tyr) 3′ UTR induce graying in rabbit

**DOI:** 10.1038/s41598-017-01727-y

**Published:** 2017-05-08

**Authors:** Yuning Song, Yuxin Xu, Jichao Deng, Mao Chen, Yi Lu, Yong Wang, Haobin Yao, Lina Zhou, Zhiquan Liu, Liangxue Lai, Zhanjun Li

**Affiliations:** 1 Jilin Provincial Key Laboratory of Animal Embryo Engineering, Jilin University, Changchun, 130062 China; 2 CAS Key Laboratory of Regenerative Biology, South China Institute for Stem Cell Biology and Regenerative Medicine, Guangzhou Institutes of Biomedicine and Health, Chinese Academy of Sciences, Guangzhou, 510530 China

## Abstract

The 3′ untranslated regions (UTRs), located at the end of mRNA molecules, are believed to play a role in RNA replication and/or protein translation. Mutations in the *tyrosinase* (*Tyr*) gene are known to cause recessive albinism in humans and other species. In this study, to test whether the CRISPR/Cas9 system works on the mutation of the UTRs regulatory region in rabbit, the 3′ UTR of the rabbit *Tyr* gene was deleted by a dual sgRNA directed CRISPR/Cas9 system. As expected, gray coat color and reduced melanin in hair follicles and irises was found in the mutated rabbit, thus increasing confidence in the association of the mutation of the *Tyr* 3′ UTR with graying in rabbit. The graying phenotype was also found in the F1 generation, suggesting that the mutated allele can be stably inherited by the offspring. Thus, we provide the first evidence that reduced melanin and graying can be caused by deletion of the *Tyr* 3′ UTR in rabbits. Additionally, CRISPR/Cas9-mediated large fragment deletions can facilitate genotype to phenotype studies of UTRs or non-coding RNAs in future.

## Introduction

Tyrosinases (*Tyr*) are essential enzymes in melanin biosynthesis and are responsible for pigmentation of skin and hair in mammals^1^. Mutations in the *Tyr* gene result in white coat color and lack of pigmentation due to absence of melanin production. Melanin biosynthesis in these mutants is disrupted at the critical first and second reactions: hydroxylation of tyrosine to L-DOPA and the oxidation of L-DOPA to DOPA-quinone^1^. Previous studies have reported that albinismis associated with a *Tyr* mutation in American mink^2^, cattle^3^, mouse^4^ and rabbit^5^. In an updated list of *Tyr* mutations, oculocutaneous albinism was also reported in humans^1^.

The rabbit tyrosinase gene (Chromosome 1: 127,562,607–127,667,237) has alength of 136 kb and is composed of five exons. In albino rabbits, a homozygous mutation (T373K) was identified that resulted in alteration of the last N-glycosylation site of the *Tyr* coding sequence^5^. Furthermore, the CRISPR/Cas9-mediated *Tyr* knockout rabbit also displayed typical albinism phenotype^6, 7^.

It is known that mammalian 5′ UTRs, which are non-coding DNA regulatory regions, mediate post- or co-transcriptional autoregulation of gene expression through direct interaction with proteins^8^. On the other hand, the 3′ UTRs direct an extensive range of alternative post-transcriptional, including regulation of mRNA decay and translation^9^, contributing significantly to gene regulation. It is well established that 3′ poly(A) tail of eukaryotic mRNAs is critical for the proper regulation of gene expression, and enhances the *in vivo* and *in vitro* translation in mammas^10^. In addition, several studies have highlighted the polyadenylation and alternative polyadenylation are important in gene expression and associated with a risk of clinical diseases^11–15^. However, all the studies were carried out *in vitro* or clinical cases, with very little studies conducted at the animal level. Therefore, there is a need to further characterize the association between the 3′ UTR and mRNA in animals.

A previous study has shown that the 5′ UTR sequence is a potentially important regulatory element for appropriate expression of the human *Tyr* gene^16^. However, very little is known about the role of 3′ UTR in *Tyr* gene expression. More recently, an insertion/deletion of 13 bp in the 3′ UTR of tyrosinase-related protein 1 (*Tyrp1*) was found in rabbits with brown coats^17^, implicating an important role of the 3′ UTR in gene expression. This study aimed to further characterize the relationship between the 3′ UTR and gene expression. Therefore, we deleted the 3′ UTR of the rabbit *Tyr* gene by dual sgRNA-directed CRISPR/Cas9 system, and the genotype to phenotype of the *Tyr* 3′ UTR were also validated in this animal model.

## Results

### Dual sgRNA-directed deletion of *Tyr* 3′ UTR in zygotes

**Figure 1:**
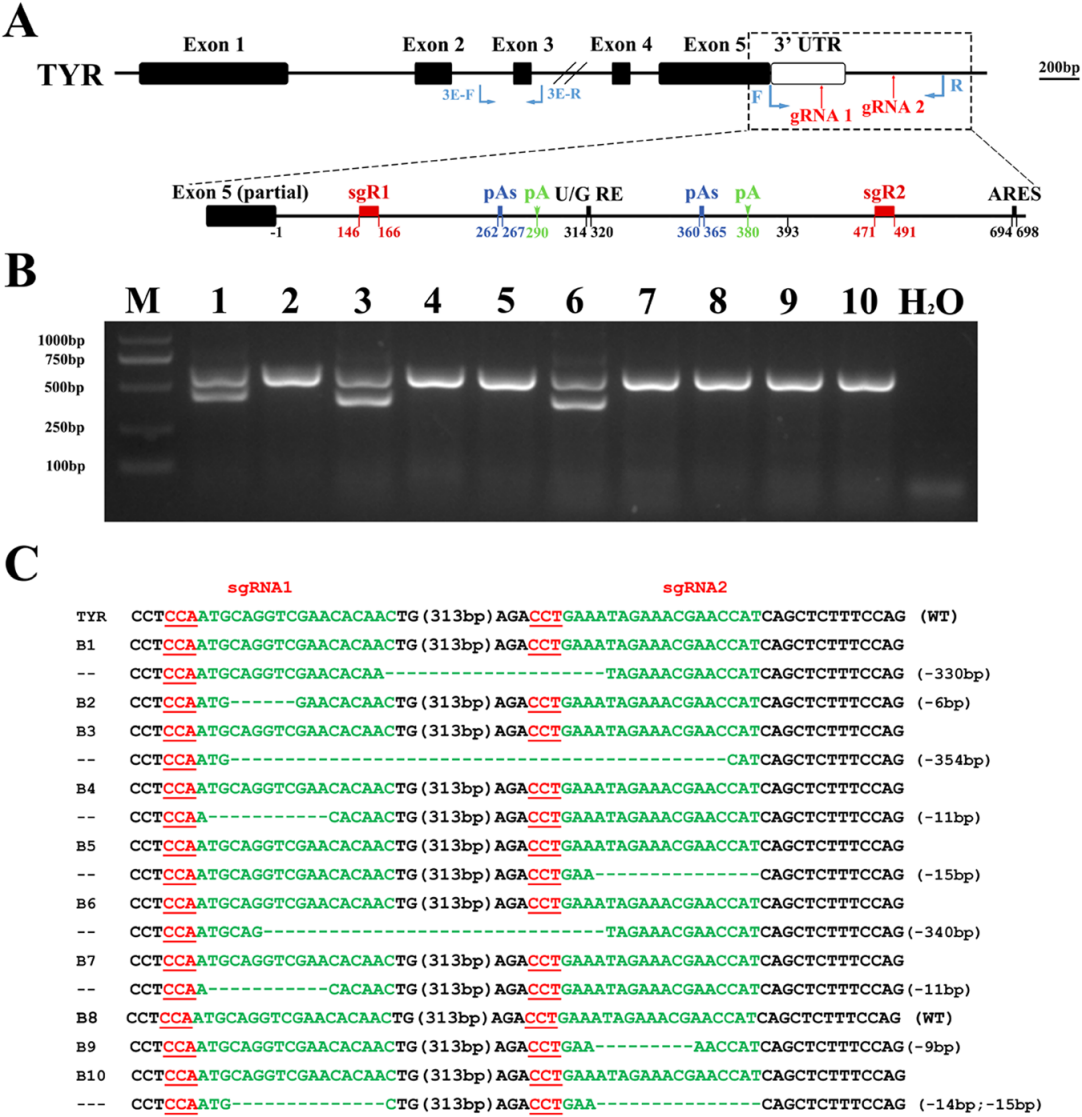
Dual sgRNA-directed deletion of the *Tyr* 3′ UTR in zygotes. (**A**) Schematic diagram of 3′ polyA tail, sgRNAs and primers targeting the rabbit TYR 3′ UTR loci. Two sgRNAs are marked in red and the primers are presented in blue. pAs: poly (**A**) addition signal; pA: poly (**A**) site; U/G RE: U/G rich area. AREs: AU-rich elements. (**B**) Mutation detection in blastocyst by PCR. M, marker; 1–10 represent different blastocysts used in this study. Original images are included “Authors’ original file for Fig. 1B”. (**C**) Mutation detection in blastocyst by T-cloning and Sanger sequencing. The WT sequence is shown at the top of the targeting sequence. sgRNA sequences are marked in green and the protospacer adjacent moti (PAM) sequences are in red with underline. WT: wild type; deletions “−”; insertion “+”.

Initially, a search for poly(A), AU-rich elements (AREs) and potential miRNA binding sites in the rabbit *Tyr* 3′ UTR was performed using online tools. As shown in Fig. 1A, we found two poly(A) sites located at 290 and 380 bp downstream of the stop codon. We then designed two guide RNAs (gRNAs) to target the 3′ UTR of the *Tyr* gene and determine the efficiency of the CRISPR/Cas9 system in zygotes. A mixture of *in vitro*-transcribed mRNA of Cas9 (100 ng/μL) and sgRNA (25 ng/μL) was injected into 21 pronuclear-stage zygotes by cytoplasm injection. Of the 17 blastocysts obtained, ten were tested by PCR amplification and sequence analysis. As shown in Fig. 1B and C, mutations were found in nine of these ten blastocysts. A 330 bp long deletion was found in B1, B3 and B6 samples, indicating that deletion of the *Tyr* 3′ UTR can be achieved in zygotes via the CRISPR/Cas9 system.

### Generation of *Tyr* 3′ UTR knockout (KO) rabbits

**Figure 2:**
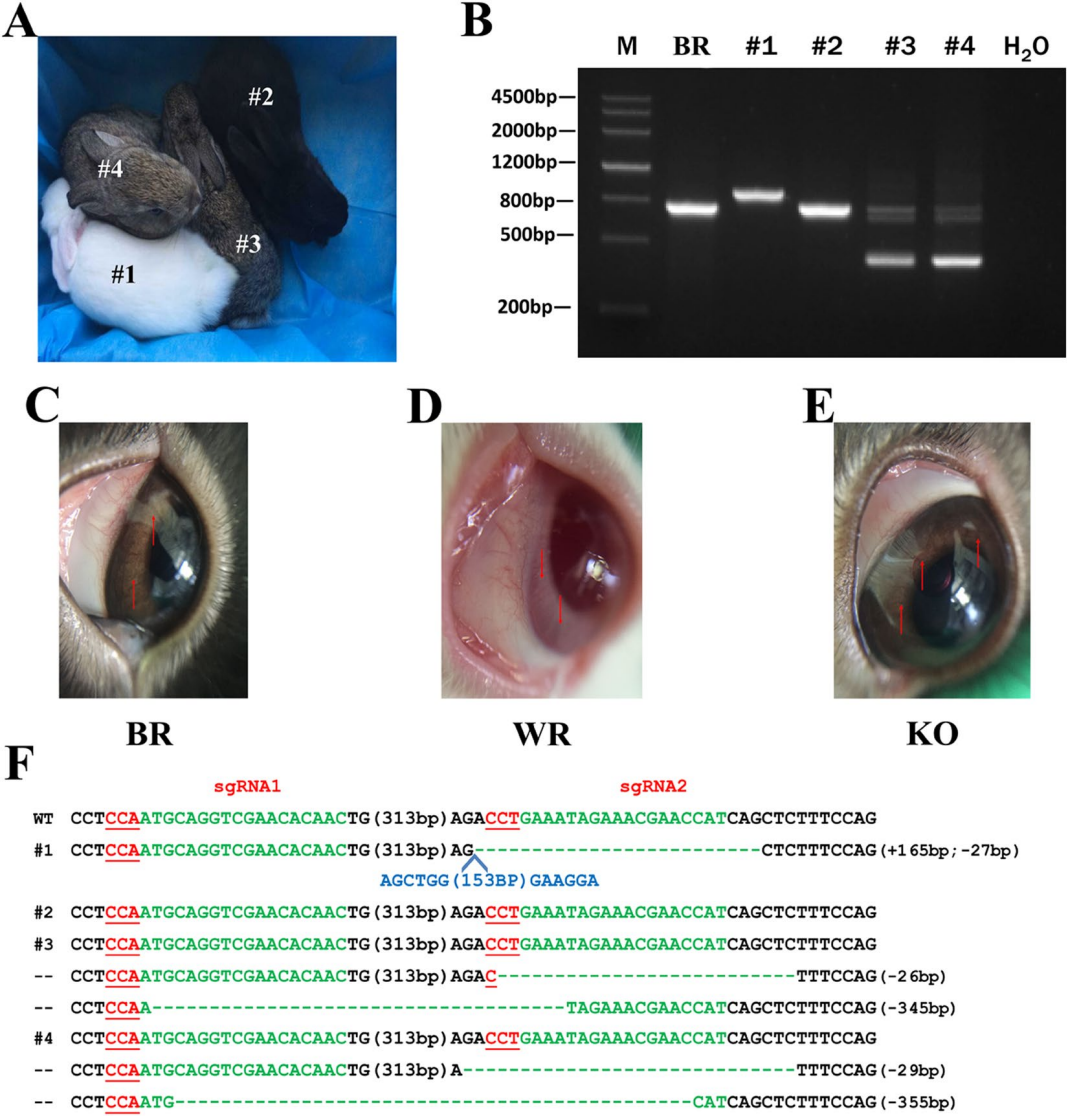
Generation of *Tyr* 3′ UTR deleted rabbit by CRISPR/Cas9. (**A**) Phenotype of *Tyr* mutant rabbits; rabbit #1, albinism; rabbit #2, black; rabbit #3 and #4, graying. (**B**) The mutation determination in founder rabbits by PCR. (**C**) The eye of black rabbit (BR). Original images are included “Authors’ original file for Fig. 2B”. (**D**) The eye of white rabbit (WR). (**E**) The eye of *Tyr* 3′ UTR deleted rabbit (KO). (**F**) Mutation detection in founder by T-cloning and Sanger sequencing. The PAM sites are underlined and highlighted in red; the target sequences are green; deletions (−) and insertions (+) are shown. WT, wild-type.

To generate *Tyr* 3′ UTR KO rabbits, 157 injected zygotes from Lianshan black rabbits were implanted into four pseudo-pregnant recipient females (Table 1). Two of the recipients carried the pregnancies to term and gave birth to four live pups (Fig. 2A). T-cloning and PCR-sequencing results revealed that a monoallelic large-fragment deletion of *Tyr* 3′ UTR was presentin pups #3 (−26 and −345 bp) and #4 (−29 and −355 bp), while an insertion (+165 bp) and a deletion (−27 bp) were detected in pup #1 (Fig. 2B and F).

**Table 1:** Generation of *Tyr* 3′ UTR KO rabbits using CRISPR/Cas9.

	gRNA/Cas9 mRNA (ng/uL)	Embryos injected	Embryos transferred	Pregnancy	Pups obtained (% transferred)	3′ UTR KO pups (% pups)	Pups with Color change
1	25/100	40	35	No			
2	25/100	45	40	Yes	2(5%)	1(50%)	0
3	25/100	40	38	No			
4	25/100	47	44	Yes	2(4.5%)	2(100%)	2

Next, we examined the coat and eye color of the 3′ UTR KO rabbits. We observed the typical albino phenotype with loss of dark pigment in the skin and eyes in rabbit #1, and graying in rabbits #3 and #4, as compared to black skin and eyes in rabbit #2 (Fig. 2C–E). The information about the genotype (T373K and 3′ UTR deletion) and skin color of F0 pups is listed in Table S3.

In addition, we observed that rabbit #1 that carried an insertion (+165 bp) showed the typical albino phenotype in the skin and eyes (Fig. 2A). Further analysis revealed that rabbit #1 had homozygous A/A genotype, while heterozygous A/C genotype was identified in rabbit #3, #4 (Table S3). The 165 bp inserted sequence was neither derived from the rabbit genome nor from the cloning vector. We therefore speculated that the non-pigmented phenotype in rabbit #1 is due to SNP (A/C) at 1118 bp of rabbit *Tyr* gene while not the insertion (+165 bp).

To test whether off-target effects occurred in these genetically modified rabbits, we screened the rabbit genome and identified five Potential off-target sites (POTs) for each sgRNA. The primers and mismatch sites are listed in Table S2. POTs were amplified by PCR from the genomic DNA of the mutant rabbits and subjected to T7E1 analysis (Fig. S1). The results revealed that none of these POTs were mutated, indicating that the sgRNAs used in this study are locus-specific.

### Reduced expression of melanin in *Tyr* 3′ UTR KO rabbits

**Figure 3:**
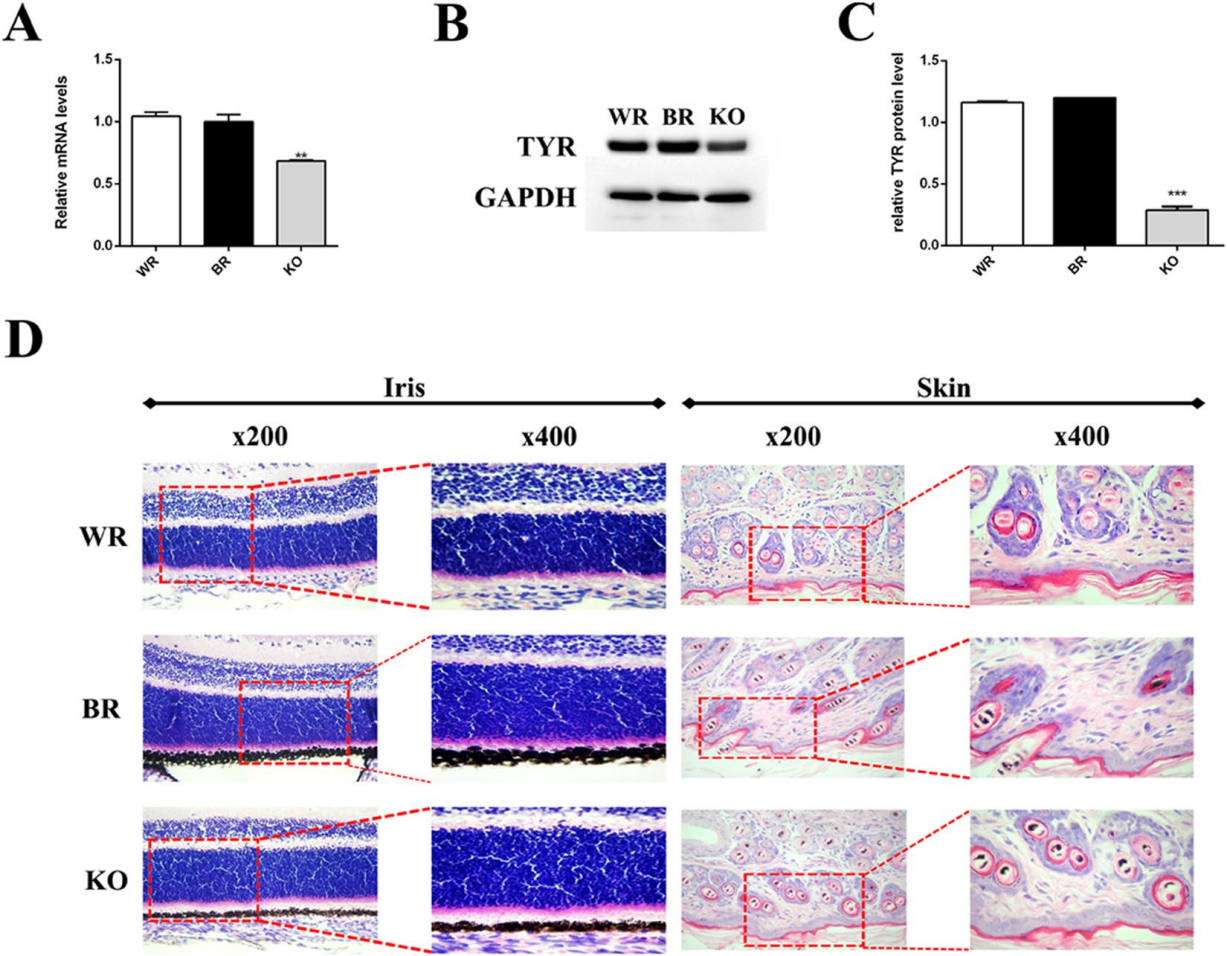
Reduced expression of melanin in *Tyr* 3′ UTR deleted rabbit. (**A**) Expression of *Tyr* gene was determined by qRT-PCR. (**B**) *Tyr* protein was determination by Western Blot. (**C**) Gray-scale analysis of the *Tyr* protein by ImageJ software. (**D**) H&E staining of the iris and skin from the WR, BR and KO rabbits. All the data are expressed as the mean ± SEM. *P < 0.005; **P < 0.001; ***P < 0.005. BR, black rabbit; WR, white rabbit; KO, *Tyr* 3′ UTR deleted rabbits.

We further examined whether deletion of the 3′ UTR lead to reduced gene expression and related phenotypes. As shown in Fig. 3A, *Tyr* mRNA level was significantly reduced in 3′ UTR KO rabbits, when compared with the New Zealand White rabbits (WR) and Lianshan black rabbits (BR). These results were also confirmed by Western blot and gray-scale analysis at the protein level (Fig. 3B,C), indicating that both *Tyr* mRNA as well as protein levels were reduced in the 3′ UTR KO rabbits. Furthermore, histological HE staining showed that melanin amounts were reduced in hair follicles and irises of 3′ UTR KO rabbits and absent in WR, when compared with BR (Fig. 3D). These results confirmed that graying and reduced melaninin hair follicles and irises was observed in the *Tyr* 3′ UTR KO rabbits.

### Heritability of *Tyr* 3′ UTR KO mutation

**Figure 4:**
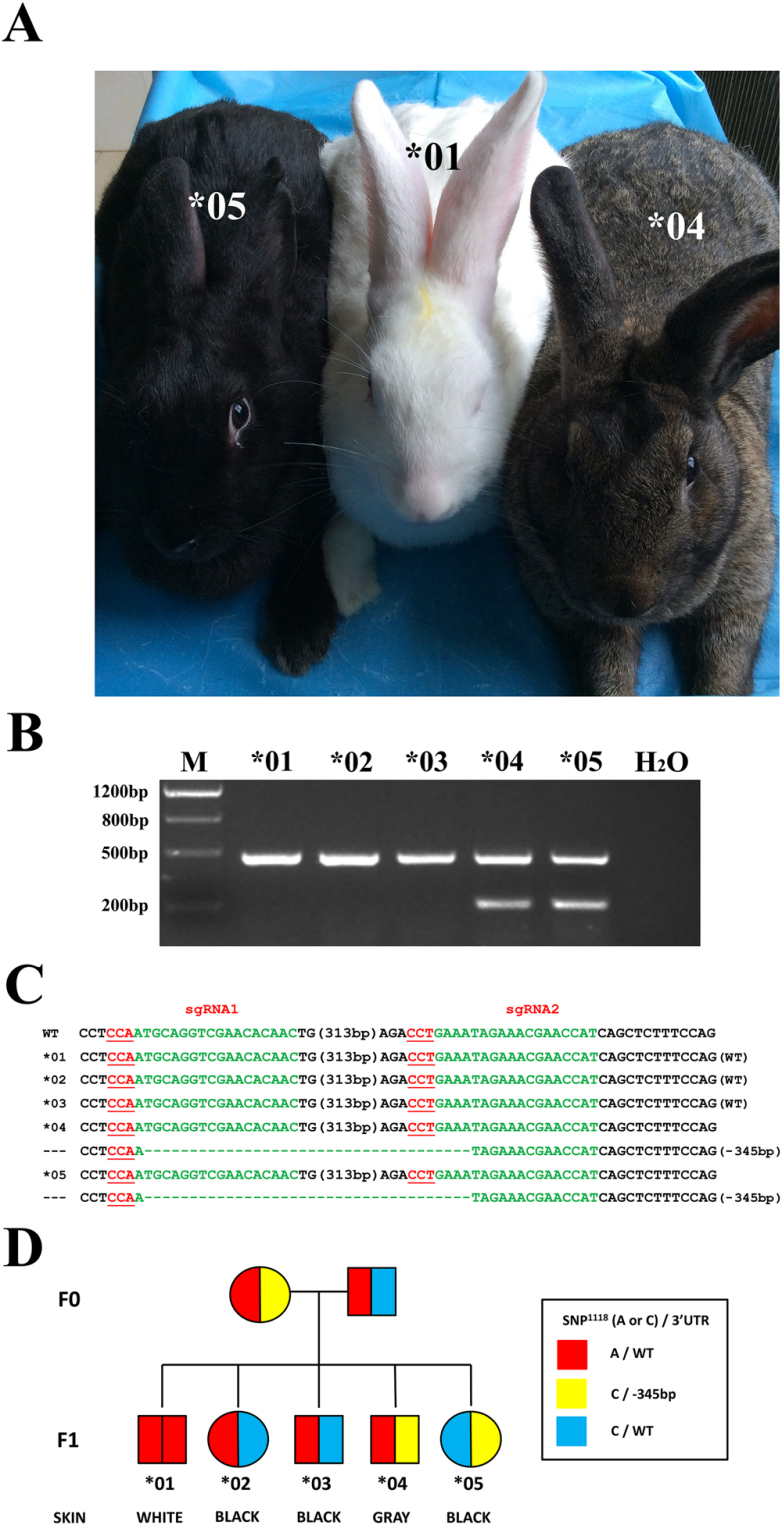
Heritability of *Tyr* 3′ UTR deleted rabbit. (**A**) The F1 pups with different skin color. (**B**) Mutation detection in F1 rabbits by PCR. The results demonstrated that the *Tyr* 3′ UTR deletions were determined in *04 and *05. M, DNA marker III. Original images are included “Authors’ original file for Fig. 4B”. (**C**) T-cloning sequence analysis of the F1 rabbits. The PAM sites are underlined and highlighted in red; the target sequences are green; deletions (−) and insertions (+) are shown. BR, black rabbit; WR, white rabbit; KO, *Tyr* 3′ UTR deleted rabbits. (**D**) A pedigree obtained from analyses in (**C**). The identical alleles are shown by the same colors. The large deletion of *Tyr* 3′ UTR in F0 is heritability transmitted to the F1 pups. SNP^1118^, the SNP sites (A/C) located in the *Tyr* gene.

Next, we then examined whether the *Tyr* 3′ UTR KO mutation and the graying phenotype could be stably transmitted to progeny. Rabbit #3 (female, 3′ UTR KO gray rabbits) was mated with Rabbit #2 (male, BR). As shown in Fig. 4A, one (*04) of five newborn rabbits showed the typical gray phenotype with reduced dark pigment in the skin and eyes. T-cloning and PCR analysis confirmed the presence of the *Tyr* 3′ UTR deletion in pups of rabbits *04 and *05 (Fig. 4B–D), demonstrating that 3′ UTR deletions are heritable and that the graying phenotype could be transmitted through the germ-line. In addition, to segregate the T373K and 3′ UTR modifications, more F1 progeny were obtained from Rabbits #3 and #2. As shown in Table S4, the rabbits carrying at least one dominant mutation (C/C, C/A) of *Tyr* are fully pigmented (*02, *03, *05, *07, *08, *11, *12), whereas the dominant mutation containing haplotype was linked to the 3′ UTR deletion and the other recessive mutant allele (A nucleotide at SNP), conferred gray coat color to the rabbits (*04, *09, *10). Similar observation was also made in the F0 generation (Table S3).

## Discussion

Previous studies have demonstrated that *Tyr* plays key roles in melanin biosynthesis and albinism^1^. We have previously described the feasibility of applying the Cas9/gRNA system to produce a large deletion (105 kb) in the *Tyr* gene, resulting in loss of pigmentation, thus leading to albinism in rabbits^6^. In this study, we presenta functional validation the *Tyr* 3′ UTR KO and albinismin rabbits. Interestingly, gray coat color and reduced melanin in hair follicles and irises was observed in rabbits with the 3′ UTR KO mutation. Therefore, we believed that the graying phenotype in these rabbits was due to targeted degradation of *Tyr* mRNAs, as has been demonstrated in previous studies^9^.

Poly(A) is presumed to play a significant role in mRNA synthesis, translation, and/or metabolism, and is potentially involved in any of the four stages of mRNA metabolism: splicing, nucleus-to-cytoplasm transport, translation, and stability^18, 19^. It has been demonstrated that the poly(A) protects mRNAs from rapid, indiscriminate degradation and also affects the half-lives of mRNA^20, 21^. In our work, we found that two poly(A) sites were destroyed in the *Tyr* 3′ UTR KO rabbits, that showed gray coat color. Here we hypothesize that destruction of the poly(A) sites affects the polyadenylation and stability of *Tyr* mRNA, resulting in lower expression of tyrosinase.

In addition, progeny from rabbit #3 appear to inherit only one of the two detected deleted alleles. Tissue mosaicism has been widely reported in genetically modified animals generated by co-injection of Cas9 mRNA and single-guide RNA (sgRNA) into zygotes^22^. In our study, the genotype of the F0 rabbits was determined from ear tissues. Therefore, we speculated that only one deleted allele was detected in the F1 progeny generated from rabbit #3 owing to mosaicism between germ cells and ear tissues.

MicroRNAs (miRNAs) are a class of endogenous non-coding small RNAs that contribute to the regulation of gene expression at the post-transcriptional level by binding to the 3′ UTR. Previous work has showed that miR-196a-2 can inhibit the expression of *Tyr* by attenuating melanin biosynthesis in the melanocytes^23^. Furthermore, six new binding sites for miRNAs have been identified, suggesting that reduced expression of *Tyr* is associated with miRNA-directed mRNA decay^9^. Unexpectedly, no miRNAs binding site was found in the rabbit *Tyr* 3′ UTR region, suggesting that the graying and reduced tyrosinase production phenotypes in the *Tyr* 3′ UTR KO rabbit is not regulated by miRNAs. The underlying molecular mechanism of this observation remains to be explored.

In summary, we provide the first evidence that a *Tyr* 3′ UTR mutation causes reduced melanin production and graying in rabbits. Moreover, functional analysis of the 3′ UTR in animal models can be achieved by dual sgRNAs directed CRISPR/Cas9 system. Our novel animal model will be beneficial to understand the relationship between the 3′ UTR region and gene expression, as well as the underlying mechanisms of genetic diseases.

## Material and Method

### Ethics statement

Rabbits involving in this experiments were New Zealand white rabbit and Lianshan black rabbit. All animal protocols were approved by the Animal Care Center and Use Committee of Jilin University. All experiments were performed according to the guidelines approved by Jilin University. New Zealand white rabbit and Lianshan black rabbit were housed under half-light condition in individual cages and were fed twice a day with commercial rabbit basic diet and water ad libitum in the Laboratory Animal Center of Jilin University.

### DNA constructs and *in vitro* transcription

The Cas9 expression construct, 3 × FLAG-NLS-SpCas9-NLS (Addgene ID 48137), was synthesized and cloned into the vector. The vector was linearized with NotI and transcribed *in vitro* according to the manufacturer’s using the mMessage mMachine SP6 Kit (Ambion, USA) and the RNeasy Mini Kit (Qiagen) to purify the mRNA.

A pair of complementary oligonucleotides encoding the 20-nt guide sequences were annealed at 95 °C for 5 min and ramped down to 25 °C to generate the dsDNA fragment, which was then cloned into the BbsI-digested pUC57-Simple vector (Addgene ID 51306) under the control of T7 promoter. The sgRNAs were transcribed using the T7 RNA Synthesis Kit (Ambion) and purified using the miRNeasy Mini Kit (Qiagen) according to the manufacturer. The concentration and quality of the synthesized mRNAs were determined by Nanodrop 2000 and agarose gel electrophoresis, respectively.

### Microinjection and embryo transfer

The protocol for microinjection of pronuclear-stage embryos has been described in detail in our published protocols^24^. Briefly, zygotes were collected from sexually matured New Zealand white rabbits, which had been superovulated by six times and a 12-h interval of intravenous injection of follicle-stimulating hormone (FSH). The rabbits were mated after intravenously injection of 100 IU human chorionic gonadotrophin (hCG). The oviducts were then flushed with 5 mL DPBS-BSA for the collection of pronuclear-stage embryos. Mixtures of *in vitro*-transcribed mRNA derived from the gRNAs (25 ng/ul) and Cas9 (100 ng/ul) were injected into the cytoplasm of pronuclear stage embryos. Then the injected embryos were transferred to embryo culture medium for 30–60 min, followed by transfer into the oviduct of the recipient mother (approximately 30–50 embryos).

### Mutation detection in embryos and pups by PCR

Each injected zygote was collected at the blastocyst stage and the DNA was extracted with embryo lysis buffer (contining1% NP40, 50 mM Tris-HCl, 150 mM NaCl, 0.5% sodium deoxycholate, 0.1% SDS) at 50 °C for 20 min and 90 °C for 5 min in a BIO-RAD PCR machine. Genomic DNA from BR, WR and KO rabbits was isolated using the TIANamp Genomic DNA Kit (TIANGEN, Beijing, China) according to the manufacturer’s instructions. PCR primers used for amplification and mutation detection are listed in Supplementary Table S1. PCR products were gel purified with TIAN gel Midi Purification Kit (TIANGEN, Beijing, China) and cloned into pGM-T (Tiangen, Beijing, China). Ten positive plasmid clones were sequenced and the sequences were analyzed by DNAMAN software.

### Off-target assay

Potential off-target sites (POTS) of the sgRNAs were predicted using the online CRISPR design tool (http://crispr.mit.edu/). The top five POTS that were selected. The primers (supplementary Table S2) were used to test whether the sgRNAs have off target mutations and the products were analyzed by both T7E1 assay and sanger sequence cloned into the pGM-T (Tiangen, Beijing, China).

### T7 endonuclease I (T7EI) assay

A T7 endonuclease I (T7EI) assay was performed as described previously^25^. Briefly, the genomic DNA of each Cas9/gRNA-injected blastocyst and its pups was amplified by PCR with primers supplied in Table S2 under following conditions: 95 °C for 5 min, 95 °C for 5 min, 95–85 °C at −2 °C/s, 85–25 °C at −0.1 °C/s, hold at 4 °C. The PCR product were digested with T7E1 (NEB M0302L) and analyzed on an ethidium bromide-stained TAE gel.

### Histology analysis

Skin and eye tissues of BR, WR and 3′ UTR KO rabbits were fixed with 4% paraformaldehyde for 48 h, embedded in paraffin wax, and slide sectioned. Skin and eye sections were stained with hematoxylin and eosin (H&E) and analyzed by microscopy (Nikon ts100).

### Real-time quantitative PCR (qRT-PCR) and Western Blotting

Total RNA from Con (n = 5) samples was isolated with TRNzol-A+ reagent (TIANGEN, Beijing, China) according to the manufacturer’s instructions. cDNA was synthesized with DNAse I (Fermentas) treated total RNA using the BioRT cDNA First Stand Synthesis Kit (Bioer Technology, Hangzhou, China). Primers used for RT-PCR are listed in Table S1. Q-PCR was performed using the BioEasy SYBR Green I Real Time PCR Kit (Bioer Technology, Hangzhou, China) with the BIO-RAD Iq5 Multicolor Real-Time PCR Detection System. The relative gene expression normalized to the GAPDH was determined by 2^−ΔΔCT^ formula. All the data of gene expression were performed three times.

For Western blotting, the eyes tissues from BR, WR and 3′ UTR KO rabbits were homogenized in 150 lL of lysis buffer. The protein concentrations were measured by the Braford method (Bio- Rad). In this study anti-TYR polyclonal antibody (1:2000; abcam) and anti-GAPDH monoclonal antibody (1:2000; Beyotime) were used as primary and internal control respectively. Images were quantified using ImageJ software (NIH) and all data are expressed as mean ± SEM.

### Statistical analyses

All data are expressed as mean ± SEM, with at least three individual determinations in all experiments. The data were analyzed by t-test using GraphPad Prism software 6.0. A probability of *p* < 0.05 was considered statistically significant.

## Electronic supplementary material

supplementary information
