# Supplementary material for: CRISPR/Cas9-mediated mutation of tyrosinase (Tyr) 3′ UTR induce graying in rabbit
Source: Sci Rep. 2017 May 8;7:1569. doi: 10.1038/s41598-017-01727-y (PMC5431497; doi:10.1038/s41598-017-01727-y)

Supplementary Information

CRISPR/Cas9-mediated mutation of tyrosinase (Tyr)3’ UTR induce graying in rabbit

Yuning Song[1#], Yuxin Xu[1#], Jichao Deng[1], Mao Chen[1], Yi Lu[1], Yong Wang[1], Haobin Yao[1], Lina Zhou[1], Zhiquan Liu, Liangxue Lai[1,2]*, Zhanjun Li[1]*

Inventory of Supplemental Information

1. Supplemental Figures

2. Supplemental Tables

3. Original file for Figures

1. Supplemental Figures

Figure S1. Off-target analysis of the 2 sgRNAs and sequence diagram of POTS in KO founders.

PCR and T7EI assays of the PCR products of candidate off-target sites for 2 sgRNAs in founder #3. No fragment was found in T7EI assays. Sequence diagram of 10 potential off-target sites for sgRNA1, sgRNA2 showing no double curve in any sequencing diagrams. Blue area represents sequencing of the POTS. Original images are included “Authors’original file for Fig. S1”.

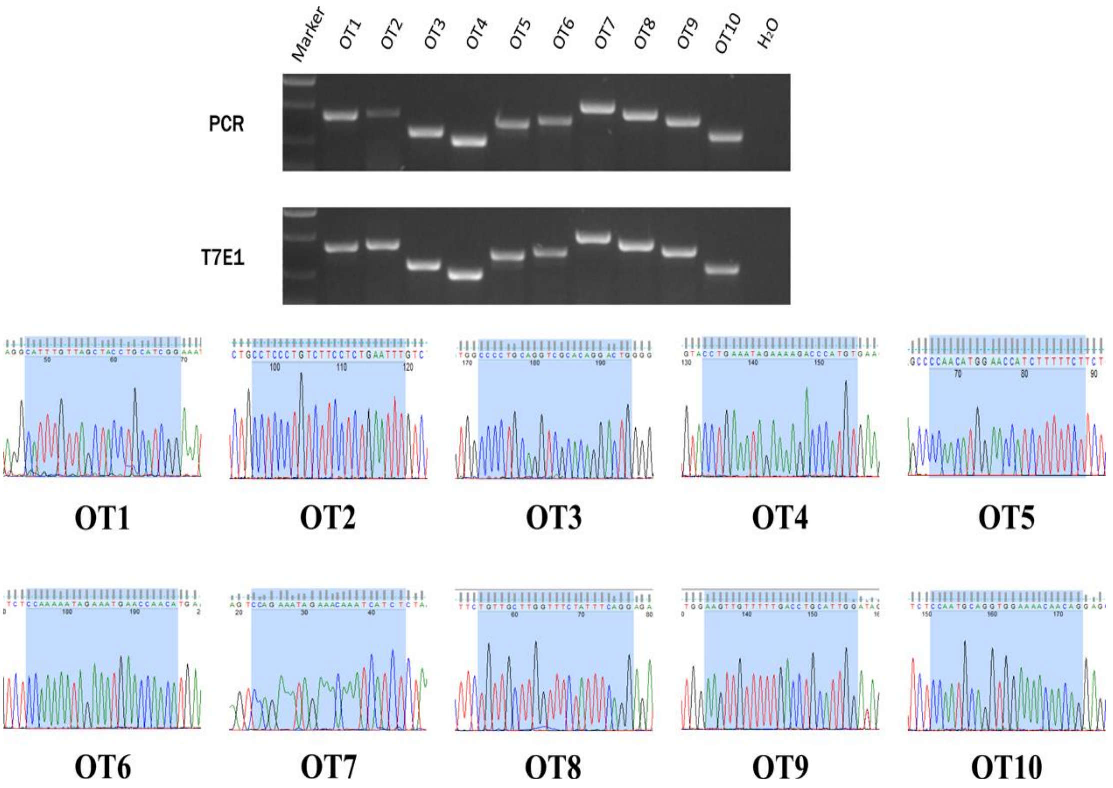

2. Supplemental Tables

Table S1: Primers used for PCR or RT-PCR .

Name	Primers	Sequence(5'-3')	Produce size (bp)
**TYR-1**	TYR-3F	CTCGTTGGAGCAGCTGTAATAG	765
	TYR-3R	CATGTCTGGAGATAGGCACTTG	
**TYR-2**	TYR-2F	TGGTTGGTAGTGTTTCTCACTT	539
	TYR-2R	GAGCACTGGCAGGTTCTATT	
**TYR-3E**	TYR-3E-F	CAGGAGAGAGAGCAAATTGGC	695
	TYR-3E-R	AGATCTGGCTGAGTCTGAGAC	
**TYR-Q**	TYR-Q-F	GGATAGCAGATGCCACTCAAA	100
	TYR-Q-R	AACGCATGGTGAAGGAGAAA	
**GAPDH**	GAPDH-F	TTCCACGGCAGGTCAAGGC	99
	GAPDH-R	GGGCACCAGCATCACCCCAC	

*TYR-1/2 was used for the PCR mutation detection in F0 and F1 KO rabbits; TYR-3E was used for the PCR of SNP analysis; TYR-Q was used to test the mRNA expression of founder rabbit.

Table S2.10 potential off-target sites examined by PCR and primers used for list.

	Potential Off Target Site	Number of mismatch	Position	PCR Primer
S1	AAGTTGTTTTTGACCTGCAT TGG	3	chr15:+70737549	F: CACCATTATTGTCCAGGGAGTT<br>R: GGTTGTCAGCTTAGGGAGTTT
	CTGTTGTTTTCCACCTGCAT TGG	3	chrUN0:-3213807	F: GCTTGGTTTGTGTGGTGTTATT<br>R: GCTGGTTTCAGCAGGAAGTA
	CATTTGTTTGCTACCTGCAT CGG	4	chr1:+37194241	F: TTGGGCAGGAGCAAATGT<br>R: CACACACACCCTAACCCTTAC
	CAGCTGTCTCCCACCTGCAT GGG	4	chr13:-37815186	F: CTACAGCTAGAGGGTGAGCATA<br>R: GAATGAGGAAACTGAGGCAGAG
	CAGTCCTGTGCGACCTGCAG GGG	4	chrUN0:-191496	F: TCCTGAAAGTGCTCCTGTC<br>R: CGAGGAGTGAGGGTGAATG
S2	ACATGGGTCTTTTCTATTTC AGG	4	chr14:-110529032	F: AGGAGAGAAGGTATTCCAGGTAG<br>R: ACTTGTGCTATTTGTCCTCCAA
	TGTTTGTTGGTTTCTATTTC TGG	3	chr17:-23711651	F: TGTGGGAGACCCAGAAGAA<br>R: AGGGAGGCAGAGAGAAGAAA
	TGTTGGTTCATTTCTATTTT TGG	3	chr9:-52227345	F: CAGAGGTTTAACCCGCTACTC<br>R: GTTGAAGGCAGACCTATTTGTTATT
	AGATGATTTGTTTCTATTTC TGG	3	chrX:-53312265	F: GTAGAAGGTGGAGTGAACAGAAA<br>R: GAAGTGTAGCAGCAGAAGACA
	TGTTGCTTGGTTTCTATTTC AGG	3	chr3:+2548445	F: TCTTTCCAAGTTTGTATCTCTCACT<br>R: ACTTCATATCTCACCACCCATTT

Table S3. The information about the genotype (T373K and 3'UTR deletion) and skin color of F0 pups

No.	Genetype	SNP at 1118	3'UTR	Skin	Gender
WR	Allele 1	A	WT	White	Female/ Male
	Allele 2	A	WT		
BR	Allele 1	A	WT	Black	Female/ Male
	Allele 2	C	WT		
#1	Allele 1	A	KO	White	Male
	Allele 2	A	KO		
#2	Allele 1	A	WT	Black	Male
	Allele 2	C	WT		
#3	Allele 1	A	WT	Gray	Female
	Allele 2	C	KO		
	Allele 3	C	KO		
#4	Allele 1	A	WT	Gray	Female
	Allele 2	C	KO		
	Allele 3	C	KO		

Table S4. The information about the genotype (T373K and 3'UTR deletion) and skin color of F1 pups

No.	Genetype (SNP at 1118 / 3'UTR)	Colour	No.	Genetype (SNP at 1118 / 3'UTR)	Colour
*01	Allele 1: A / WT<br>Allele 2: A / WT	White	*07	Allele 1: A / WT<br>Allele 2: C / WT	Black
*02	Allele 1: A / WT<br>Allele 2: C / WT	Black	*08	Allele 1: C / WT<br>Allele 2: C / KO	Black
*03	Allele 1: A / WT<br>Allele 2: C / WT	Black	*09	Allele 1: A / WT<br>Allele 2: C / KO	Gray
*04	Allele 1: A / WT<br>Allele 2: C / KO	Gray	*10	Allele 1: A / WT<br>Allele 2: C / KO	Gray
*05	Allele 1: C / WT<br>Allele 2: C / KO	Black	*11	Allele 1: A / WT<br>Allele 2: C / WT	Black
*06	Allele 1: A / WT<br>Allele 2: A / WT	White	*12	Allele 1: C / WT<br>Allele 2: C / KO	Black

3. Original file for Figures

3.1 Authors' original file for figure 1B

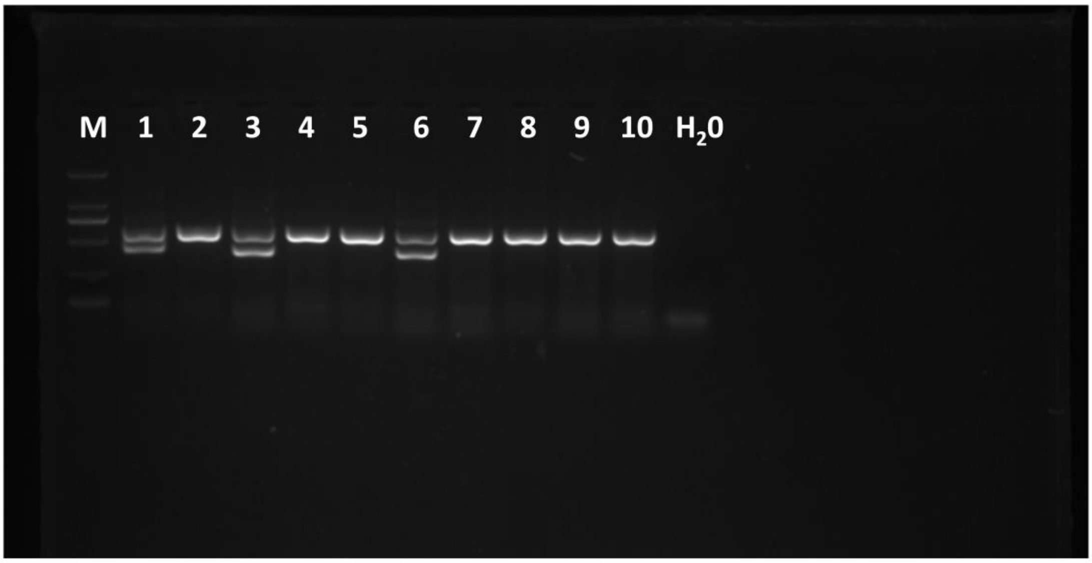

3.2 Authors' original file for figure 2B

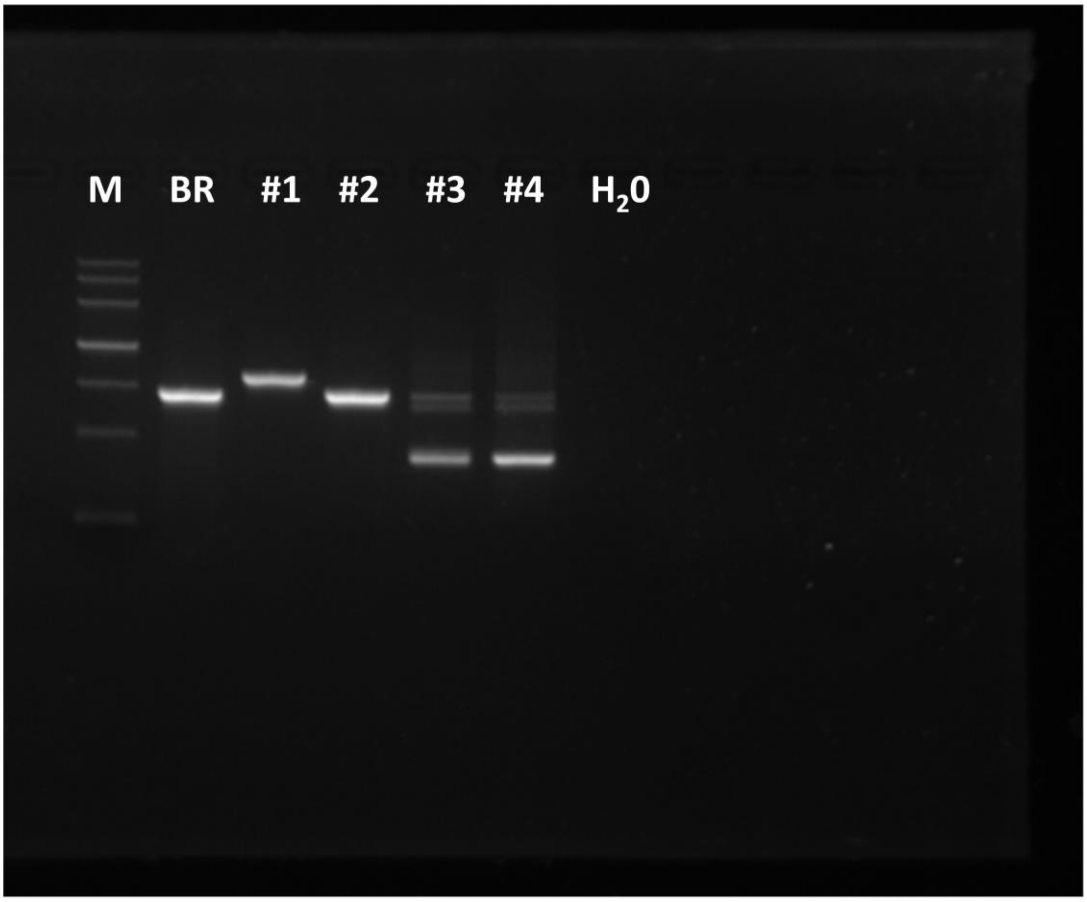

3.3 Authors' original file for figure 4B

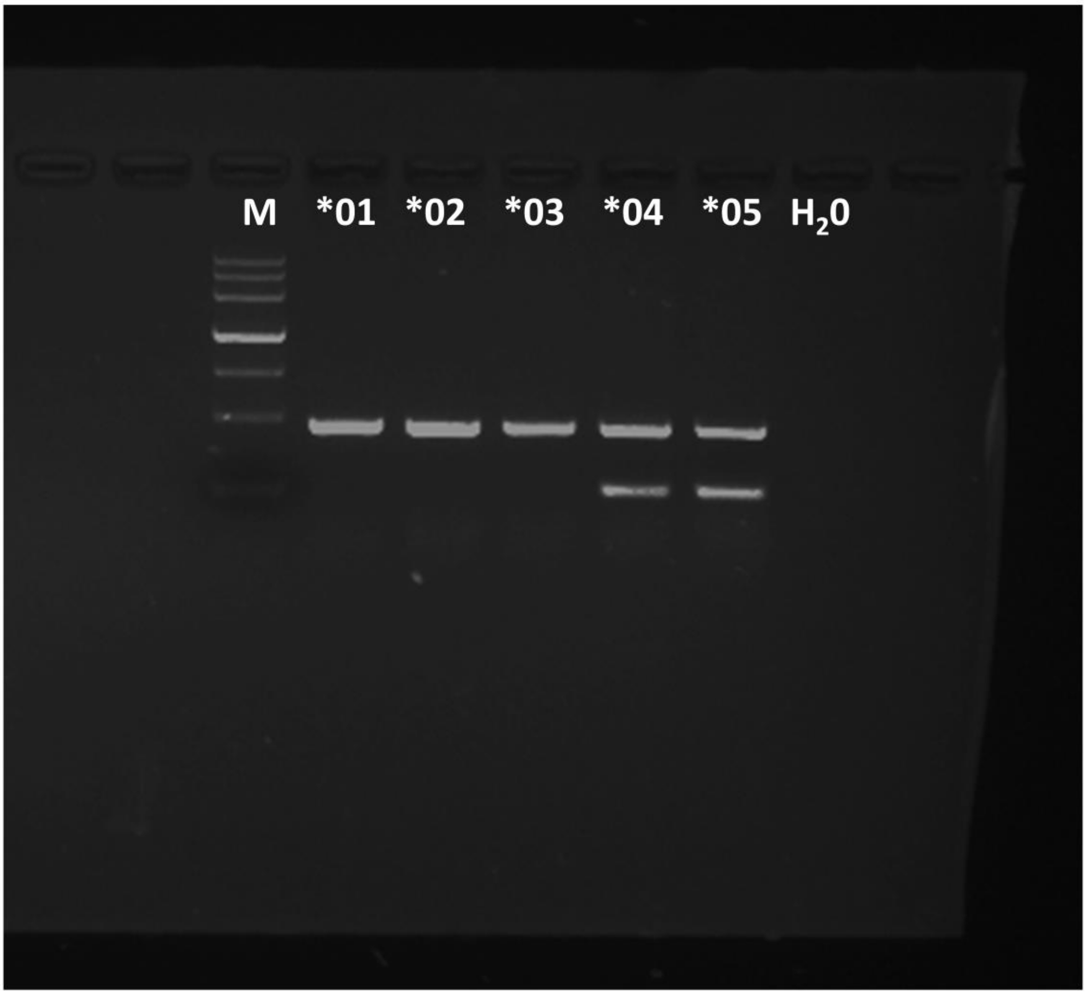

3.4 Authors' original file for figure S1

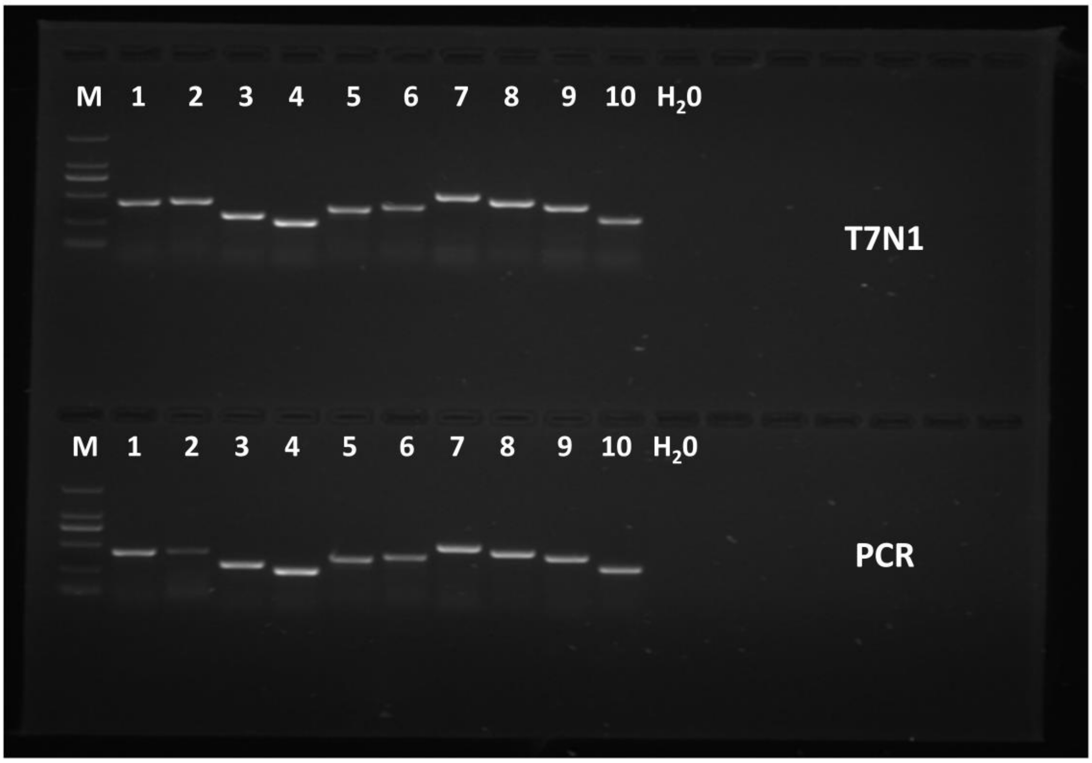

Supplement: Supplementary file 1 — supplementary information [file 41598_2017_1727_MOESM1_ESM.pdf]
